# A single point mutation in precursor protein VI doubles the mechanical strength of human adenovirus

**DOI:** 10.1007/s10867-017-9479-y

**Published:** 2017-12-15

**Authors:** Mariska G. M. van Rosmalen, Glen R. Nemerow, Gijs J. L. Wuite, Wouter H. Roos

**Affiliations:** 1 Natuur- en Sterrenkunde and LaserLaB, Vrije Universiteit Amsterdam, Amsterdam, The Netherlands; 2 Department of Immunology and Microbiology, the Scripps Research Institute, La Jolla, CA USA; 3 Zernike Instituut, Rijksuniversiteit, Groningen, The Netherlands

**Keywords:** Adenovirus, Protein VI, Atomic force microscopy, Nanoindentation, Mechanical properties, Force spectroscopy

## Abstract

Viruses are extensively studied as vectors for vaccine applications and gene therapies. For these applications, understanding the material properties of viruses is crucial for creating optimal functionality. Using atomic force microscopy (AFM) nanoindentation, we studied the mechanical properties of human adenovirus type 5 with the fiber of type 35 (Ad5F35) and compared it to viral capsids with a single point mutation in the protein VI precursor protein (pVI-S28C). Surprisingly, the pVI-S28C mutant turned out to be twice as stiff as the Ad5F35 capsids. We suggest that this major increase in strength is the result of the DNA crosslinking activity of precursor protein VII, as this protein was detected in the pVI-S28C mutant capsids. The infectivity was similar for both capsids, indicating that mutation did not affect the ability of protein VI to lyse the endosomal membrane. This study highlights that it is possible to increase the mechanical stability of a capsid even with a single point mutation while not affecting the viral life cycle. Such insight can help enable the development of more stable vectors for therapeutic applications.

**Electronic supplementary material:**

The online version of this article (10.1007/s10867-017-9479-y) contains supplementary material, which is available to authorized users.

## Introduction

Human adenoviruses (AdV) cause acute respiratory, gastrointestinal, and ocular infections and are therefore the object of intense study. In addition, AdV is increasingly being studied as a vector for gene therapy and vaccine applications. [1–8]. Exploring the material properties of AdV will help to create a more stable AdV vector for such therapeutic applications and will support the development of targeted antiviral strategies. Several studies have focused on the mechanics of AdV [9–12]. These studies revealed how the maturation transition increases the genome-induced pressure inside the capsid [9] and how the flexibility of AdV is linked to successful infection [12]. In the latter study, opposite effects on capsid stability were observed between (i) αv integrin binding, which facilitates virus endocytosis and uncoating, and (ii) α-defensins, which restrict endosome escape and infection. Upon binding of integrin, the stiffness at the vertex region of the capsid is decreased, thereby stimulating uncoating of the viral capsid. In contrast, binding of defensin HD5 resulted in an increased stiffness at the vertex region, thus preventing the release of the penton base and release of the membrane lytic protein VI from the interior of the capsid.

Human AdV is a non-enveloped double-stranded DNA virus of about ~90 nm in diameter. The pseudo *T* = 25 icosahedral capsid consists of 240 trimeric hexon capsomers, 12 penton capsomers located at the 12 fivefold axes of the icosahedral symmetry (vertex region), and 12 fiber proteins protruding from the penton bases. In addition, there are four minor capsid cement proteins (IIIa, VI, VIII, and IX), five genome-associated proteins (V, VII, μ, and terminal protein (TP)) and adenoviral protease (AVP), the latter of which is responsible for converting precursor proteins from the immature virion into their mature (cleaved) form [13–16]. Cleavage of these proteins during maturation results in decreased condensation of the DNA, preparing for a highly cooperative DNA release. This DNA alteration results in an increase of the internal pressure and consequently an increase in the spring constant of the capsid [9]. Additionally during maturation, the capsid is primed for uncoating by destabilizing the penton bases, creating a less stable capsid [10, 11]. Uncoating or disassembly starts when the fiber binds to the primary receptor on the cell surface followed by internalization [17–19]. In the endosome, the pentons are released together with some internal components including protein VI, which has been shown to induce a pH-independent disruption of the endosomal membrane [20, 21]. Then the partially uncoated capsid traffics to the nuclear pore complex [22, 23] where the final uncoating takes place, allowing the viral genome to enter the nucleus [24].

Nanoindentation by atomic force microscopy (AFM) is an emergent technique to characterize the mechanical properties of artificial as well as natural nano-sized structures [25–28]. In addition, by using AFM imaging, one also obtains topographical information on the sample allowing both morphological and mechanical analyses of the same nano-structured sample. This combined imaging and force spectroscopy technique is increasingly being used to study the mechanics of single viral nanoparticles during the different stages of the viral life cycle as well as the influence of individual proteins [12, 29–37]. Here we focus on the role of a multifunctional capsid protein known as protein VI, in the elasticity and stability of the AdV capsid. The precursor form of protein VI is involved in the stabilization of the immature capsid by interacting with the inner cavity of each hexon [38, 39] and the C-terminal 11 amino acids of this molecule accelerates the activity of the AVP [40]. Together with its membrane disrupting capacity, this shows that protein VI plays an important role in the infectivity process of AdV. We performed AFM nanoindentations to determine the stiffness of human AdV type 5 with the fiber of type 35 (Ad5F35) and this same viral capsid with a single point mutation in the precursor protein form of protein VI. In this mutant, the serine at residue 28 is replaced by a cysteine that is near the N-terminus of protein VI, resulting in mutant pVI-S28C particles. This mutation was generated as part of a larger series of protein VI mutants used to define the functions of this cement protein. We selected the pVI-S28C mutant for AFM studies because we anticipated that its propensity to undergo interchain disulfide bonding might shed some light on the involvement of protein VI in the maturation process and the infectivity of AdV. This mutant also provided an opportunity to study the role of protein VI on the elasticity and stability of the capsid.

## Materials and methods

### Adenovirus

Human AdV type 5 with fiber of type 35 (Ad5F35) and this same viral capsid with a single point mutation, in which, the serine at residue 28 is replaced by a cysteine at the N-terminal end, in the precursor protein form of protein VI (mutant pVI-S28C) were prepared as described previously by Moyer et al. [41]. Both viruses have a deletion of the E1 region and are thus incapable of replication in nearly all mammalian cells lacking this region. The purified Ad5F35 and pVI-S28C mutant virus particles were analyzed by SDS-PAGE, followed by Simply Blue (Life Technologies, Carlsbad, California, USA) staining (6 μg virus/lane).

### Infectivity plaque assay

To determine the infectivity, 293β5 (American Type Culture Collection, Manassas, Virginia, USA) cells were seeded in six-well plates at 4 × 10^5^ cells per well in duplicate. When a confluency of 70–80% was reached, they were infected with either Ad5F35 or pVI-S28C mutant (0.25 vp/cell) and rocked for 2 h at 37 °C. After removal of the inoculum, the cells were washed once with PBS. Then, the cells were carefully overlaid with 4 ml/well of equal parts of Avicel 2.4% (RC-581) and 2× EMEM (BioWhittaker, Waltham, Massachusetts, USA) supplemented with 2× penicillin/streptomycin, 2× L-glutamine and 10% Fetal Bovine Serum (FBS). The plates were incubated for 72 h at 37 °C, followed by scanning with a Typhoon 9410 imager (GE Healthcare Life Sciences, Chicago, Illinois, USA). The plaques were quantified with ImageJ. A more detailed protocol is given by [42].

### Thermal stability assay

The thermal stability of virions was measured by the accessibility of the viral DNA to a fluorescent intercalating dye, TOTO-1 (Molecular Probes, Waltham, Massachusetts, USA) as previously described by [43]. In short, 100 μg/ml of Ad5F35 or pVI-S28C was incubated with 60 nM TOTO-1 and the fluorescence emission of TOTO-1 was monitored as a function of temperature in an ABI Prism 7900HT real-time PCR machine (Applied Biosystems, Waltham, Massachusetts, USA) programmed to measure fluorescence (λ_ex_ 488; λ_em_ 540) every 2.5 °C between 20 and 70 °C. Samples were equilibrated at each temperature for 2 min prior to fluorescence measurement.

### Atomic force microscopy

For imaging and nanoindentation experiments the AdV capsids were immobilized onto a hydrophobic glass surface prepared as described previously [44]. In short, first the glass slides were cleaned by overnight incubation in an ethanol–water bath saturated with potassium hydroxide followed by thoroughly washing with MilliQ water and drying by air. The cleaned glass slides were incubated overnight in hexamethyldisilazane vapor to make them hydrophobic. The sample was diluted in DX10/10 buffer (40 mM Tris, 500 mM sodium chloride, 2% (wt/vol) sucrose and 1% (wt/vol) mannitol) to a final concentration of ~10 ng/μl and incubated on the hydrophobic glass surface at a minimal volume of 100 μl for approximately 15 min at room temperature, before imaging started in buffer solution.

Images and nanoindentation curves were obtained with an AFM from Nanotec Electronica (Madrid, Spain), operated in jumping mode [45] at room temperature. Olympus (Tokyo, Japan) OMCL-RC800PSA rectangular, silicon-nitride cantilevers with a nominal tip radius of 15 nm and a nominal spring constant of 0.05 N/m were used. The average imaging force was 105 ± 11 pN. Obtained images were processed and analyzed using the WSxM software. All reported heights obtained from the images are corrected for the scanning force according to:

$$ {\mathit{\mathsf{Height}}}_{\mathit{\mathsf{real}}}={\mathit{\mathsf{Height}}}_{\mathit{\mathsf{measured}}}+\frac{{\mathit{\mathsf{F}}}_{\mathit{\mathsf{imaging}}}}{\kappa_{\mathit{\mathsf{virus}}}} $$

where *F*_imaging_ is the imaging force and *κ*_virus_ is the spring constant of the capsid. For nanoindentations, we zoomed in on individual capsids and positioned the tip at the center of the capsid. Five consecutive nanoindentation cycles were performed on each capsid with a loading speed of ~90 nm/s. The obtained force–distance curves were analyzed with a home-build Matlab program. In short, force–distance curves were transferred into force–indentation curves by subtracting the glass curve. The first linear part of the force–indentation curve, up to 11 nm or until the critical point, was used to determine the spring constant. Additionally, a close-up image of the capsid after indentation was obtained to determine the amount of damage inflicted by the indentations. The stated error, unless specified otherwise, is the standard error of the mean. Student’s* t* test (two-sample unequal variance with two-tailed distribution) was used to determine differences between groups.

## Results

### pVI-S28C mutant

**Fig. 1:**
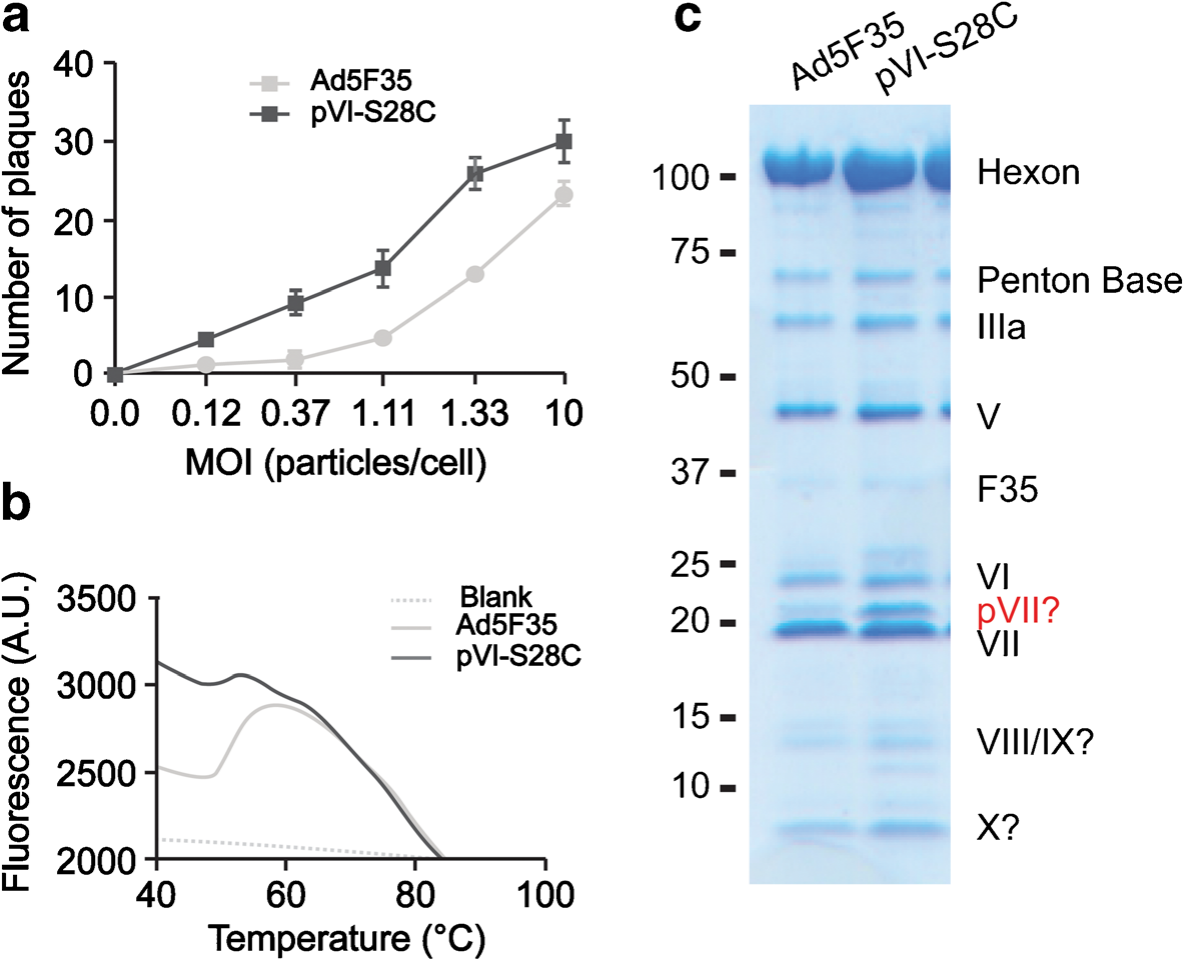
Infectivity, thermal stability, and protein content of Ad5F35 versus pVI-S28C capsids.** a** The infectivity rate of both Ad5F35 and pVI-S28C capsids are comparable.** b** The thermal stability of the pVI-S28C capsids is increased compared to Ad5F35 capsids.** c** SDS page gel electrophoresis analysis of the known capsid proteins. The pVI-S28C mutant and Ad5F35 virions had a similar complement of capsid proteins, except the pVI-S28C mutant contained an additional ~22-kDa protein that might correspond to precursor protein VII (pVII) and more smaller proteins

We compared the infectivity and the thermal stability of the pVI-S28C mutants with that of the Ad5F35 virions. The infectivity of the pVI-S28C mutant as determined by plaque assay (Fig. 1a) appeared to be similar to Ad5F35. The pVI-S28C mutant had only a slight (~2-fold) increase in infectivity compared to the Ad5F35, suggesting that the mutation did not significantly affect the ability of protein VI to lyse the endosomal membrane. The thermal stability of pVI-S28C and Ad5F35 virions was determined by TOTO-1 fluorescent dye incorporation thermal stability experiments (Fig. 1b). The Ad5F35 displayed the characteristic temperature-induced dye uptake at 42–45 °C. In contrast, the virions containing the pVI-S28C mutation did not display this pronounced dye uptake profile, indicating a change in the thermal stability of the pVI-S28C mutant particle. Thus, the infectivity was similar; however, the thermal stability was higher for the pVI-S28C mutant particles. To determine whether the pVI-S28C mutant virions contained a different array of the known capsid proteins than Ad5F35 virions, we subjected purified virions to SDS page gel electrophoresis analyses. In general, the pVI-S28C mutant and Ad5F35 virions had the same complement of capsid proteins, although the pVI-S28C mutant virus contained an additional ~22-kDa protein, which might correspond to precursor protein VII (pVII), which would be consistent with the recent findings of Dai et al., who report that pVII is associated with the inner hexon cavity [39] (Fig. 1c). This suggests that the maturation process was somewhat incomplete in the case of the pVI-S28C particles.

### Morphology of the capsids

**Fig. 2:**
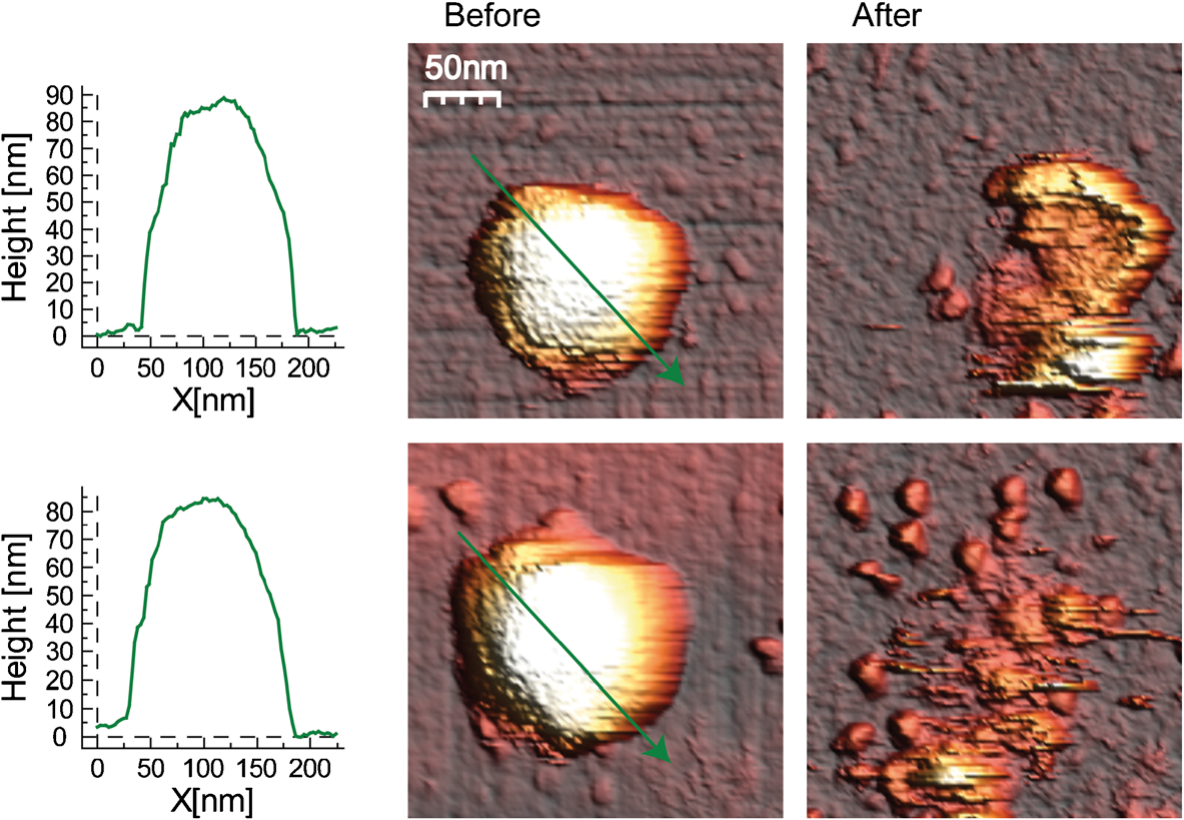
Structure of AdV capsids before and after indentation. Two examples are represented in a row with on the* left* the height profiles along the arrows of the AdV capsids represented in the 3D images in the* middle*. The measured height of the icosahedral capsids is ~85–90 nm. The images on the* right* contain the same capsids but after indentation, representing the damage inflicted by the indentation. The* top* capsid is partially broken, while some of the sides are still standing with some debris in the middle. The* bottom one* is more severely damaged and completely disassembled into smaller substructures

We used AFM for imaging of AdV capsids to determine their morphology before and after nanoindentation. In total, we imaged 120 AdV capsids of which 35 were Ad5F35 capsids and 85 pVI-S28C mutant capsids. Figure 2 shows two examples of topographical AFM images of the Ad5F35 capsids before and after indentation. Before indentation, all capsids were intact, e.g., cases where a capsid was missing a penton capsomer or had other visible damage were not included for nanoindentation analyses. Less than 5% of the capsids had to be excluded because of such visible damage. After nanoindentation, many different structures were observed, ranging from almost complete capsid disassembly into hexon and penton capsomeres of ~11 nm (Fig. 2, bottom) to a partial collapse of the capsid (Fig. 2, top). Occasionally, we observed capsids that presented with a hole in their center while the remaining sides of the capsid were still intact. Overall, before indentation, all capsids were intact, while after indentation all capsids showed different levels of damage.

**Fig. 3:**
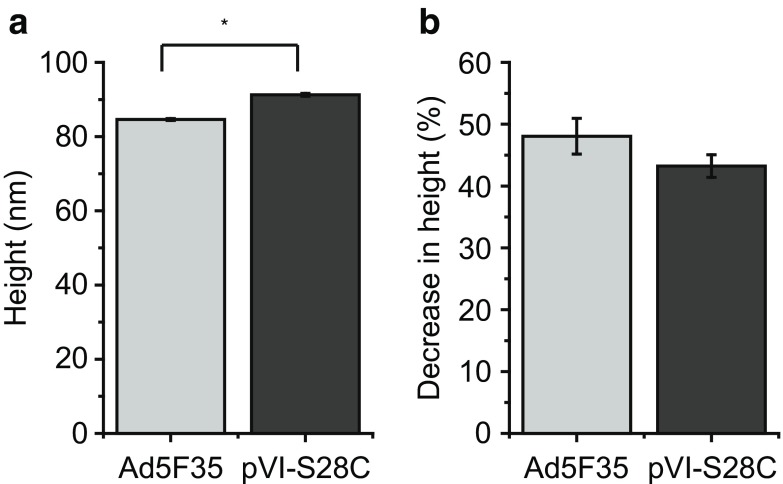
Average height before and after nanoindentation of the AdV capsids.** a** Height of Ad5F35 capsids (*n* = 35) before indentation is 84.6 ± 0.3 nm, which is significantly lower than the height of the pVI-S28C mutant (*n* = 85) 91.3 ± 0.4 nm (*Student’s *t* test < 0.01).** b** Upon nanoindentation the height of Ad5F35 capsids decreased by 48 ± 3% and the pVI-S28C mutant decreased 43 ± 2%. All heights obtained for the intact capsid are corrected for scanning force as stated in the Materials and methods section

To roughly compare the level of damage inflicted by nanoindentation, we determined the height before and the maximal height of the remaining structure after nanoindentation. Before indentation the average height of the Ad5F35 was 84.6 ± 0.3 nm, which is comparable to the diameter based on the AdV crystal structure [14]. The height of the pVI-S28C mutant capsids was 91.3 ± 0.4 nm, which is a ~10% increase compared to the Ad5F35 capsids (Fig. 3a). This increase might be due to the lack of full maturation resulting from potential crosslinking of hexon to pVII in the pVI-S28C capsids. For classification of the damage inflicted by nanoindentation, we calculated the percentage of the decrease in height for each type of viral capsid. In case of Ad5F35, the average decrease in height was determined to be ~48 ± 3%, which is within error the same as the 43 ± 2% decrease observed for the pVI-S28C mutant capsids (Fig. 3b).

### Mechanical properties of AdV capsids

**Fig. 4:**
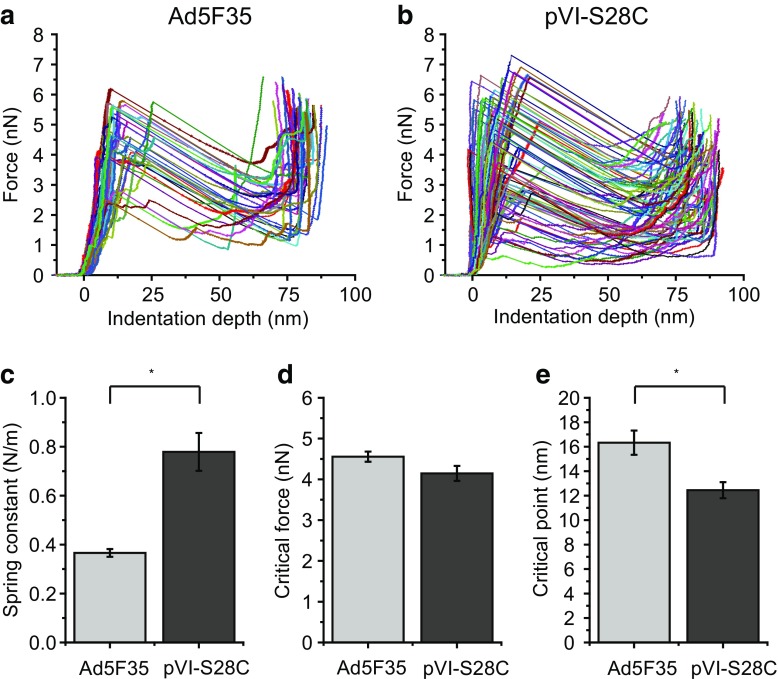
Nanoindentation results of Ad5F35 and pVI-S28C mutant capsids. Force–indentation curves of indentations performed on Ad5F35 (**a**) and pVI-S28C mutant (**b**) capsids. There is a linear response up to the critical point, at which irreversible damage occurs to the capsid and which is characterized by a significant drop in force. The force increases again upon contact between the AFM tip and the remaining structure and the underlying stiff glass surface. Spring constant (**c**), critical force (**d**) and critical point (**e**) of both Ad5F35 and pVI-S28C mutant capsids (*Student’s* t *test < 0.01)

To explore the precise impact of the pVI-S28C mutation on tensile strength of viral capsids, we determined the spring constant, critical force, and critical point of both capsids. The spring constant is calculated from the slope of the initial linear part of the force–indentation curve. The force–indentation curves of all capsids are shown in Fig. 4a-b. We observed a significant increase of the spring constant for the pVI-S28C capsids compared to the Ad5F35 capsids. For the Ad5F35, we determined a spring constant of 0.37 ± 0.02 N/m while the pVI-S28C mutant had a spring constant of 0.78 ± 0.08 N/m. The critical force at which the capsids deform irreversibly and break was comparable, 4.6 ± 0.1 nN for Ad5F35 and 4.2 ± 0.2 nN for pVI-S28C. A difference in spring constant but similar critical forces means that the corresponding indentation depth at the critical point should be different. Indeed, for Ad5F35 we determined a critical point of 17 ± 1 nm while 12.5 ± 0.7 nm was observed for pVI-S28C. Thus, we obtained a twofold increased spring constant, a similar critical force and a significantly increased critical point for the pVI-S28C mutant capsids compared to the Ad5F35 capsids (Fig. 4c-e).

### Capsid orientation

**Fig. 5:**
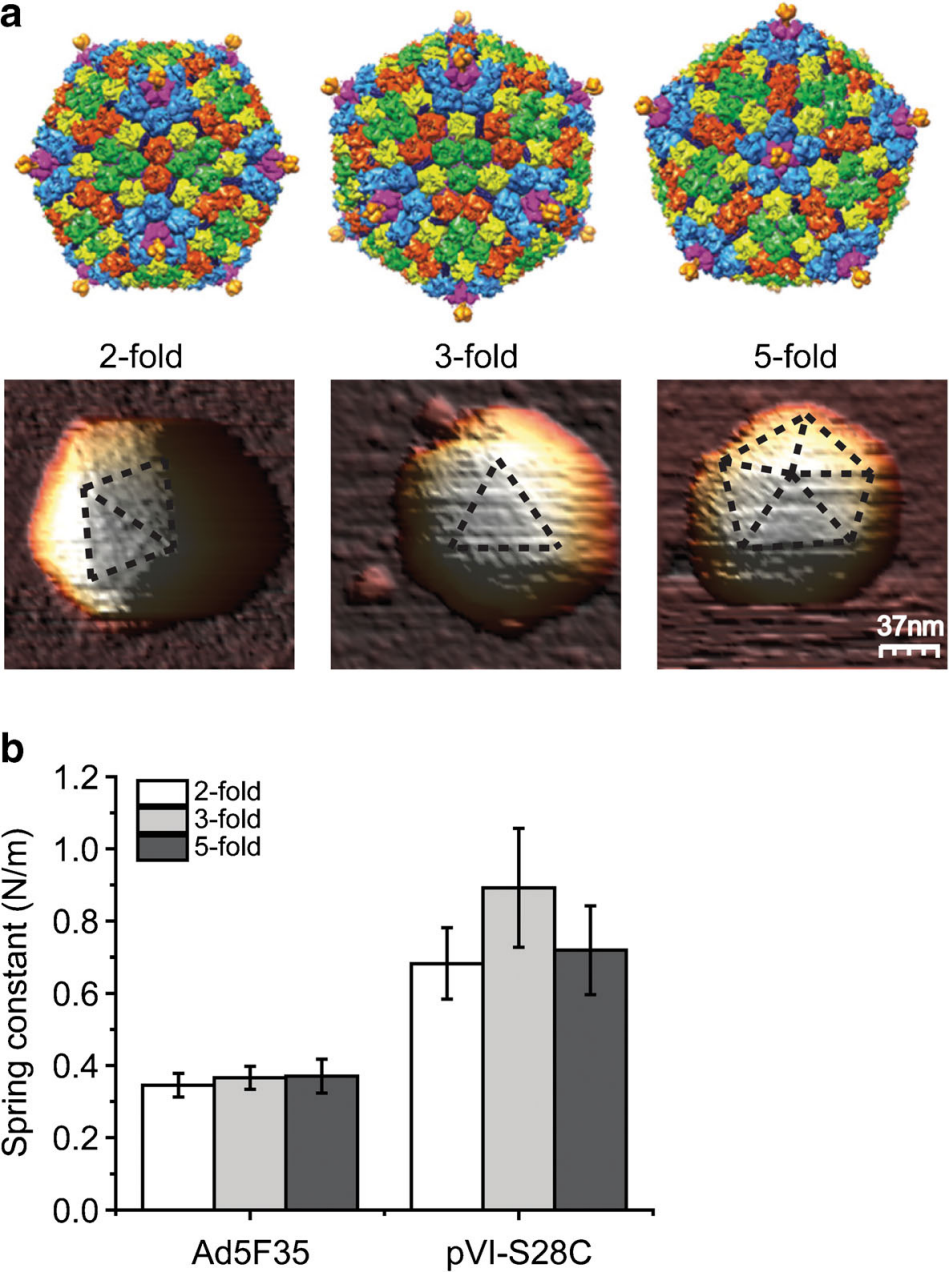
Structural morphology of intact AdV capsids and their spring constant based on orientation.** a** The *top row* shows surface renderings of AdV capsids based on the crystal structure at 3.8-Å resolution (Protein Data Bank [PDB] code, 1VSZ) representing the three different orientations of the icosahedral capsid, with twofold, threefold, and fivefold symmetry. The* colored hexon capsomers* represent the four unique hexon trimers and the penton capsomers are shown in* magenta* with the protruding fibers in* orange*. The* bottom row* shows 3D topological AFM images of Ad5F35 capsids in the corresponding three orientations indicated by the* dashed lines*.** b** Spring constants of AdV capsids obtained at different orientations. The indentation response for each virus type is independent of orientation

AdV has an icosahedral capsid consisting of 20 triangular facets, 12 vertices, and three principal axes of symmetry, meaning that the capsid can adsorb in three different orientations onto the glass surface. Figure 5a shows examples of immobilization on the three symmetry axes. The system can exhibit a twofold orientation when the capsid is lying on the edge of two triangular facets, a threefold orientation when the capsid is positioned with one triangular facet onto the surface, or a fivefold orientation when the capsid is adhered to the surface with a vertex. These orientations can be distinguished in the images recorded prior to the nanoindentation. When we correlated the nanoindentation results with the absorption geometry, we observed a similar mechanical response regardless of orientation (Fig. 5b and Supplementary Fig. 1). We determined a spring constant of 0.35 ± 0.03 N/m for the twofold orientation of the Ad5F35 capsids together with 0.37 ± 0.03 and 0.37 ± 0.05 N/m for the threefold and fivefold orientations, respectively. For pVI-S28C capsids, we obtained spring constants of 0.7 ± 0.1, 0.9 ± 0.2 and 0.7 ± 0.1 N/m for twofold, threefold, and fivefold, respectively. Thus, there is a significantly different spring constant observed between the Ad5F35 and pVI-S28C capsids, but no significant difference in spring constant between the orientations of the Ad5F35 or of the pVI-S28C capsids.

## Discussion

We compared the infectivity, thermal stability, and mechanical properties of the Ad5F35 capsid and this same capsid containing the pVI-S28C mutation to explore the involvement of protein VI in the maturation process and infectivity and the effect of this mutation on the elasticity and stability of the AdV capsid. Regarding capsid maturation, we determined whether the mutant virions contained a different array of the known capsid proteins than the non-mutant virions. In general, both capsids had the same complement of capsid proteins, although the pVI-S28C mutant virions contained an additional ~22-kDa protein and possibly also smaller proteins. This ~22-kDa protein is expected to correspond to pVII, which suggests that the maturation process was incomplete. The pVI-S28C mutation, however, did not seem to affect the cleavage of pVI, which is cleaved by AVP at both the C-termini and N-termini. The cleaved C-terminus of pVI is shown to be involved in stimulating the activity of the AVP [40]. Our results suggest the S28C mutation located at the N-terminus of pVI somehow affects the ability of AVP to cleave pVII, and possibly other precursor proteins. However, the exact mechanism underlying this remains unclear. One of the main questions to be answered is how AVP is able to gain access to the amino terminal region of pVI, which is partially buried inside the hexon trimer [39].

The infectivity of the pVI-S28C mutant is similar to unaltered Ad5F35, suggesting that the mutation did not affect the ability of protein VI to lyse the endosomal membrane. On the contrary, the previously studied pVI-G48C mutation also located on the N-terminal end did impair protein VI release due to dimerization of the cleaved protein VI, which affected the endosomal membrane disruption and thus virus infectivity [46]. Also, another pVI mutation, pVI-G33A, which is located immediately prior to the AVP cleavage site, affected the ability of AVP to cleave pVI and impaired membrane disruption and virus infection as well as capsid assembly [41]. In another unrelated AdV study, it has been shown that immature *ts1* AdV capsids also exhibit a decreased infectivity as a result of an impaired uncoating; they become trapped in the endosome due to a lack of exposure of protein VI [19]. This suggests that despite the pVI-S28C mutation, pVI was successfully cleaved by the AVP during maturation, which is in agreement with our observed SDS page gel electrophoresis results of the array of present proteins in the capsids and the unaltered infectivity.

A previous study showed an increased thermal stability for the immature *ts1* AdV capsid compared to the mature wild-type AdV capsid [11]. This would be in agreement with our expectation that our pVI-S28C mutant would be a not fully matured capsid due to the presence of pVII and therefore would have an increased thermal stability compared to the Ad5F35 capsids. This might suggest a possible role of pVII, and maybe other present precursor proteins in the pVI-S28C capsids, in determining the capsids thermal stability [11, 38]. Another possible explanation for the increased thermal stability of the pVI-S28C mutation could be an altered affinity of the inter-hexon interactions. The N-terminus part of pVI has been shown to bind intimately at the base of peripentonal hexons and is involved in the assembly process of the capsid [46–48]. It has been speculated that maturation may alter the hexon interaction [41]. Since the N-terminus of pVI is tightly bound to the hexons, a mutation in the N-terminus might affect the inter-hexon interactions, which in the case of our mutant pVI-S28C could cause increased thermal stability. Thus, the increased thermal stability of the pVI-S28C mutant virions could result from the presence of precursor proteins due to an incomplete maturation process or altered inter-hexon interactions.

In mechanical fatigue experiments, it was shown that the immature *ts1* virions break more gradually due to their condensed core compared to wild-type virions, which are prone to release their decondensed, loosely packed DNA for efficient infection [10, 49]. Both the pVI-S28C and Ad5F35 capsids show a comparable relative decrease in height due to nanoindentation, which suggests that both capsids contained decondensated DNA ready for infection. Thus the presence of pVII in the pVI-S28C capsids did not result in condensation of the DNA despite pVII being associated with genome interactions [13–15, 50]. Most likely, the level of cleaved VII, and the other proteins associated with genome interactions, was sufficient for decondensation of the DNA during the maturation process. Thus, the DNA inside the pVI-S28C capsids was decondensated due to maturation, which explains the similar relative decrease in height obtained for the Ad5F35 and pVI-S28C mutant capsids.

Initially, we expected the pVI-S28C capsids to be somewhat immature, given the presence of an additional ~22-kDa protein that likely corresponds to pVII. Thus, we expected a decreased spring constant for the pVI-S28C mutant capsids since Pérez-Berná et al. [11] and Ortega-Esteban et al. [9] showed a decreased spring constant for the immature AdV5 *ts1* mutant compared to the mature AdV5 capsids. As mentioned above, however, the similar relative decrease in height of both studied capsids leads us to speculate that the DNA inside both capsids was decondensated and therefore a comparable spring constant for both capsids was expected. Contrary to our expectations, we recorded a twofold increase in spring constant for the pVI-S28C mutant virions. Based on the literature, we can speculate that the presence of the pVII protein might cause this increase. pVII is shown to crosslink with the viral DNA, thus the decondensated DNA might be crosslinked to the viral shell, causing a further increase in stiffness compared to the Ad5F35 capsids. For other viruses, it has been shown that not only the presence of the genome but also the interaction between genome and capsid influences the mechanical properties of the viral capsid [37, 51]. Alternatively, an altered affinity of the inter-hexon interactions could also explain the major increase in strength. Since the N-terminus of pVI is tightly bound to the hexons [41, 46–48], a mutation in the N-terminus might affect the inter-hexon interactions, which in the case of our mutant, pVI-S28C, would stiffen the capsid structure.

Several previous studies reported nanoindentation experiments on AdV5 capsids. In contrast to the study of Snijder et al. [12], we obtained a slightly lower spring constant of 0.37 ± 0.02 N/m at the threefold orientation of Ad5F35 compared to the earlier reported 0.43 ± 0.01 N/m. Moreover, the reported orientational dependence of the spring constants was not found in the current study, which could be due to differences in determining the point of indentation between these studies. The reported break force of 4.6 ± 0.2 nN in Ref. [12] is similar to the force of 4.56 ± 0.12 nN we found here. A study by Ortega-Esteban et al. [9] also reported nanoindentation experiments on wild-type AdV5 and determined a spring constant of 0.56 ± 0.02 N/m and a breaking force of 5.0 ± 0.1 nN, which both are higher than our observations. In another study from the same lab, Pérez-Berná et al. [11] reported a spring constant of 0.46 ± 0.2 N/m and a breaking force of 3.3 ± 0.2 nN for mature AdV5 virions. The differences in spring constant might be caused by a difference in selection of the particles for nanoindentation, as suggested by the authors. The differences in break force might be caused by the difference in loading rate used for the nanoindentation experiments, ranging from 55 to 150 nm/s. Snijder et al. [52] showed an increased break force for a higher loading rate for CCMV, HK97, and Phi29 capsids. Also, the tip radius has been shown to influence the mechanical response. In a recent publication on nanoindentation experiments of fluid nanovesicles, a larger tip radius was shown to produce an earlier stiffening response of the vesicles [53]. Taken together, this shows that a direct comparison of spring constants and break forces of different studies has to be done with care.

## Conclusions

We used unaltered Ad5F35 and this same capsid containing a pVI-S28C mutation to explore the influence of protein VI on maturation and infectivity. We obtained a similar infectivity for both particles, suggesting that the pVI-S28C mutation did not affect one of the major known functions of protein VI, lysing of the endosomal membrane. However, we did detect the presence of pVII, indicating that the AVP was somewhat affected by the mutation. This might explain the increased thermal stability we observed for the pVI-S28C mutants since this would be consistent with the thermal stability of a *ts1* immature AdV capsid. Furthermore, we obtained a twofold increased spring constant for the pVI-S28C mutant compared to Ad5F35. This could also be explained by the presence of pVII, since pVII is known to crosslink the DNA, which is likely to lead to a stiffer response. While our study sheds new light on the complex influence of protein VI on maturation and infection, the results also reveal that further experiments on protein VI mutants are necessary to form a complete picture of the role and effects of this important adenoviral capsid protein.

## Electronic supplementary material

ESM 1 (DOCX 71 kb)
