# Supplementary material for: A single point mutation in precursor protein VI doubles the mechanical strength of human adenovirus
Source: J Biol Phys. 2017 Dec 15;44(2):119–32. doi: 10.1007/s10867-017-9479-y (PMC5928017; doi:10.1007/s10867-017-9479-y)

**Supplementary Information**

A single point mutation in precursor protein VI doubles the mechanical strength of human adenovirus

*Mariska G. M. van Rosmalen^1^, Glen R. Nemerow^2^, Gijs J.L. Wuite^1,*^ and Wouter H. Roos^3,*^*

*^1^* Natuur- en Sterrenkunde and LaserLaB, Vrije Universiteit Amsterdam, The Netherlands

*^2^* Department of Immunology and Microbiology, the Scripps Research Institute, La Jolla, CA, USA

*^3^* Zernike Instituut, Rijksuniversiteit Groningen, The Netherlands

^*^ Corresponding author emails: [g.j.l.wuite@vu.nl](mailto:g.j.l.wuite@vu.nl), [w.h.roos@rug.nl](mailto:w.h.roos@rug.nl)

**Supplementary Fig.** 1 Height and mechanical properties of the Ad5F35 and the pVI-S28C mutant capsids subdivided in orientation. a) Ad5F35 has a height of 84.6±0.4, 84.0±0.5 and 85.7±0.7 nm for 2-fold, 3-fold and 5-fold respectively. Mutant S28C has a height of 91.8±0.4, 90.1±0.9 and 92.0±0.6 nm for 2-fold, 3-fold and 5-fold respectively . b) The percentage of decrease in height as a results of nanoindentation is 50±5 45±7 and 52±9 % for Ad5F35 and 43±3, 43±3 and 44±3 % for pVI-S28C for 2-fold, 3-fold and 5-fold respectively. c) Ad5F35 has a critical force of 4.4±0.2, 4.6±0.3 and 4.3±0.5 nN for 2-fold, 3-fold and 5-fold respectively. The critical force of mutant pVI-S28C for 2-fold, 3-fold and 5-fold are 4.3±0.3, 4.2±0.4 and 3.9±0.3 nN respectively. d) The critical point of the Ad5F35 capsids are 16±1, 19±3 and 15±2 nm and 12±1, 12±1 and 12±1 nm for pVI- S28C mutant capsids for respectively 2-fold, 3-fold and 5-fold. In none of the cases there is a significant difference in between the orientations of the Ad5F35 of pVI-S28C capsids.


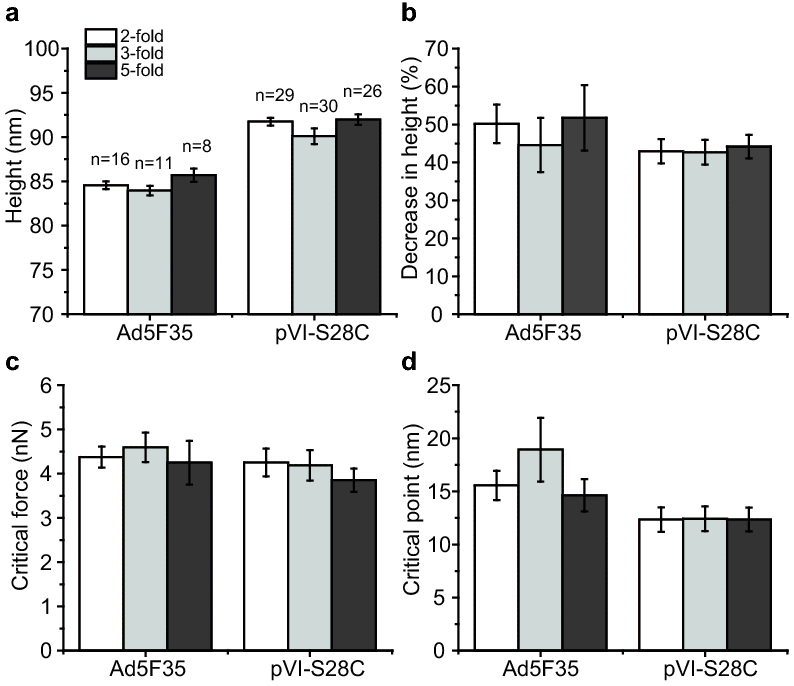

Supplement: Supplementary file 1 — (DOCX 71 kb) [file 10867_2017_9479_MOESM1_ESM.docx]
